# Spectrofluorimetric Approach for Quantification of Cyclizine in the Presence of its Toxic Impurities in Human Plasma; in silico Study and ADMET Calculations

**DOI:** 10.1007/s10895-022-02897-3

**Published:** 2022-03-03

**Authors:** Michel Y. Fares, Nada S. Abdelwahab, Ghada M. El-Sayed, Maha A. Hegazy, Maha M. Abdelrahman

**Affiliations:** 1 Pharmaceutical Chemistry Department, Faculty of Pharmacy, Nahda University, Sharq El-Nile, Beni-Suef, 62511 Egypt; 2 Pharmaceutical Analytical Chemistry, Faculty of Pharmacy, Beni-Suef University, Alshaheed Shehata Ahmad Hegazy St, Beni-Suef, 62514 Egypt; 3 Analytical Chemistry Department, Faculty of Pharmacy, Cairo University, Kasr El-Aini Street, Cairo, 11562 Egypt

**Keywords:** Cyclizine, Impurities, Spectrofluorimetric, Human plasma, ADMET

## Abstract

Cyclizine (CYZ); an antiemetic compound; is widely misused for its euphoric or hallucinatory effects, either by oral or intravenous routes. The concomitant abuse of CYZ among addicted adolescents contributes to neuromuscular disorders that are life-threatening. Consequently, with the company of 1-Methylpiperazine (MPZ) and diphenylmethanol (DPM, Benzhydrol) as pharmacopoeia-reported CYZ impurities, a novel spectrofluorimetric assay for the detection of CYZ, has been established either in human plasma samples or in its parenteral formulation. The native fluorescence of CYZ has been investigated under various conditions. Different parameters affecting relative fluorescence intensity of CYZ including diluting solvent, surfactant, plasma protein solvent, and pH were studied and optimized. The linearity obtained between the fluorescence intensity at emission wavelength 350 nm after excitation at 244 nm and the corresponding CYZ concentrations was in the range 10–1000 ng/mL for measurement of CYZ either in pure form or in human plasma samples, with a appropriate correlation coefficient (r = 0.9999) and 3.10 ng/mL as the limit of detection and 9.41 ng/mL as the limit of quantitation. The suggested procedure was created and validated in accordance with ICH guidelines for quantification of CYZ either in its pure form or its dosage form, and FDA guidelines for the assay of CYZ in human plasma. Finally, in silico study and ADMET predictions were conducted for the studied drug impurities to estimate their pharmacokinetic behaviors. The results showed that both CYZ impurities have higher cellular permeability and maximum tolerated doses, DPM has higher BBB and CNS permeability than MPZ, while MPZ exceeds DPM in total clearance and volume of distribution.

**Graphic Abstract:**

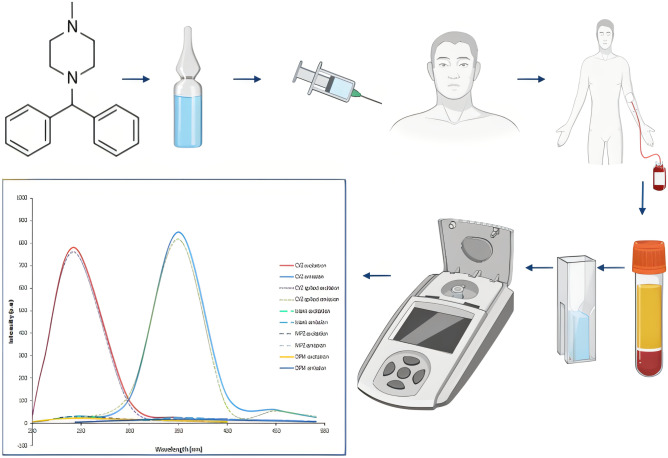

## Introduction

**Fig. 1:**
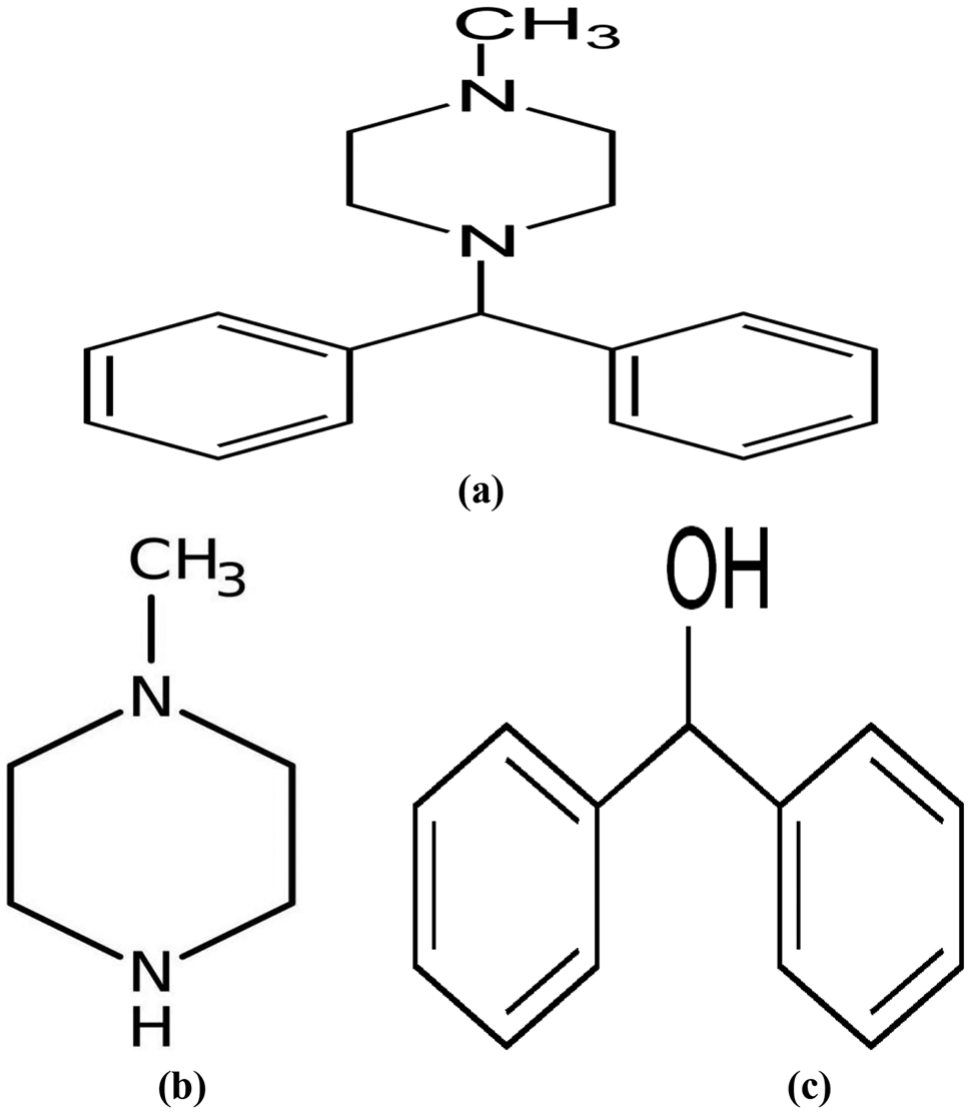
Chemical structures of cyclizine (**a**), methylpiperazine (**b**) and diphenylmethanol (**c**)

Cyclizine (CYZ), 1-benzhydryl-4-methylpiperazine or 1-(diphenylmethyl)-4-methylpiperazine, is a piperazine derivative which has been competently indicated for the preclusion and therapy for nausea and vomiting linked to motion sickness Fig. 1 [1]. It is an antihistaminic drug with sedative effect besides having H1 antagonist activity with central anti-muscarinic action [2]. Typically CYZ is administered post-operatively as an anti-emetic drug, but it is considered to have potential for addiction and hallucinogenic effects [3].

Lately, teenagers or patients with cancer in the inpatient unit of major cancer centers have misused CYZ in large doses intravenously (IV) [4]. Many cases of CYZ dependency were found in people with conventional types of recreational drug abuse, but long-term opiates are reported in patients with chronic pain of uncertain origin. Abuse of CYZ has been documented for more than 40 years, but these concerns are not commonly recognized [5]. There are many side effects of overdose and illegal use of CYZ. The most frequently described symptoms were confusion, hallucinations, disorientation, tremors, drowsiness, dysarthria, chest pain, ataxia, seizures, as well as lead to suicide [6].

Cyclizine dependence also reported in patients with complex nutritional problems [5]. According to British Pharmacopoeia (BP), it has two impurities; impurity A named 1-Methylpiperazine (MPZ) and impurity B named Diphenylmethanol (DPM) or benzhydrol Fig. 1. Both impurities are harmful, toxic, and dangerous to human bodies when CYZ parenteral formulations are given intravenously. They result in respiratory failure as well as damage to a variety of target organs [7, 8].

A literature review revealed that several approaches to the determination of CYZ have been identified, either in the formulation of raw materials or pharmaceuticals, either alone or in conjunction with other medications. These recorded analytical approaches include non-aqueous titration [1], spectrophotometric [9–11], TLC-densitometric [12–14], HPLC [15–20], capillary zone electrophoretic [21], and potentiometric [22–25] methods. Neither in its pharmaceutical formulation nor in biological fluids, have no spectrofluorimetric methods for determining CYZ been published.

In analytical chemistry, fluorescence is the most widespread and useful form of photoluminescence [26]. Spectrofluorimetric analysis was conducted because of its high level of sensitivity, selectivity, simplicity, and low cost, which can scan a large number of samples in a short amount of time [27].

Pharmacokinetic parameters like absorption, distribution, metabolism, excretion and toxicity (ADMET) profiling of compounds were studied by the pkCSM ADMET descriptors algorithm protocol [28]. In silico study means that this study conducted on computer or via computer simulation. It has many advantages in lowering the need for costly laboratory work and clinical trials for either drug discovery or ADMET predictions. In silico methods in ADMET-associated predication have many applications in estimate aqueous solubility, pKa, blood- brain barrier and CNS permeation [29].

This work aims to establish a new, selective, simple and highly sensitive spectrofluorimetric approach for the analysis of CYZ in the presence of its toxic impurities or in biological matrices. The developed method was effectively employed for analysis of CYZ in its parenteral preparation and human plasma which allow its application for routine quality control analysis dependent on its natural fluorescence. Additionally, prediction the pharmacokinetic parameters of CYZ in human plasma and its two impurities were conducted by using in silico study and ADMET predictions.

## Material and Method

### Apparatuses

The Agilent Cary Eclipse Fluorescence Spectrofluorimeter (USA) was used, which equipped with a 150 W xenon flash lamp and a 1 cm quartz cell. The excitation and emission monochromator slit widths were both set to 10 nm. Cary Eclipse scan application software version 1.2 was used to set up the spectrofluorimeter.

### Materials and Reagents

#### Chemicals and Solvents

All of the solvents and chemicals used in this study were analytical grade, and none of them were purified further.

Monopropylene glycol (MPG), sodium dodecyl sulfate (SDS), tween 80, polyethylene glycol 400 (PEG 400), acetone, ethyl acetate, disodium hydrogen phosphate, hydrochloric acid, sulfuric acid, and phosphoric acid were obtained from El-Nasr Pharmaceutical Chemicals Co., (Abu-Zabaal, Cairo, Egypt). Acetonitrile, ethanol, and methanol were of HPLC grade (Fisher, Loughborough, UK). Deionized water obtained from (SEDICO pharmaceuticals Co., 6^th^ October City, Egypt).

#### Pure Standards

Pure standard CYZ was gently given by Amoun Pharmaceuticals Company (El Obour city, Cairo, Egypt), the purity of the substance was determined using the reported HPLC method [20] and it was found to be 99.89 %. 1-Methylpiperazine (MPZ) and diphenylmethanol (DPM, Benzhydrol) were bought from Sigma-Aldrich (St. Louis, MO), with certified purities of 99.91 and 99.98, respectively.

#### Pharmaceutical Formulation

Emetrex^®^ ampules (Batch No. 182541 A) labelled to contain 50 mg/mL of CYZ. It is produced by Amoun Pharmaceuticals Company (El Obour city, Cairo, Egypt) and was purchased from local drug market.

### Solutions

#### Standard Solutions

CYZ, MPZ, and DPM stock standard solutions were prepared in ethanol at a concentration of 1.0 mg/mL. 0.10 g of the compounds was weighed into 100-mL volumetric flasks, dissolved in 50 mL ethanol, and ethanol was added until the mark was reached. To obtain a 10.0 g/mL working standard solution of CYZ, correctly transfer 0.5 mL from the CYZ stock standard solution (1.0 mg/mL) into a 50-mL volumetric flask, and then fill to the mark with ethanol.

#### Quality Control (QC) Samples

Samples of QC of CYZ working solutions were prepared at three concentration levels: low, medium, and high as (50, 400, 800 ng/mL). Quality control samples were held at -20°C in the refrigerator. Before the analysis, they were all melted at room temperature.

#### Pharmaceutical Formulation Solution

Emetrex^®^ ampules (Batch No. 182541 A) labelled with a 50 mg/mL CYZ concentration. It is manufactured by Amoun Pharmaceuticals Company (El Obour city, Cairo, Egypt), and the Emetrex^®^ ampules used in this study were obtained from the local pharmacy.

### Procedure

#### Spectral Characteristics

To establish the excitation and emission wavelengths for maximum intensity in each solvent, the native fluorescence activity was first evaluated in a pre-scan mode. After that, determine the emission intensity at maximum ƛ by setting the wavelength of maximum excitation intensity.

#### General Analytical Procedure

Measured volumes of CYZ working solution of 0.1–10 µL were diluted to a final volume of 10 mL by acetonitrile. Finally; the fluorescence intensity of the obtained solutions was captured at a wavelength of emission of 350 nm after excitation at 244 nm. By following the same analytical methods but omitting the studied compound, blank measuring was accomplished. The final concentration of CYZ in ng/mL was graphed against the obtained fluorescence intensity to create the calibration curve, and the corresponding linear regression equation was developed.

#### Procedure for Pharmaceutical Formulations

As stated below the pharmaceutical formulation solution section, within the linearity range, various sample solutions were formulated and diluted with acetonitrile to get the anticipated concentrations within the linearity range of CYZ following the general analytical procedure. The concentrations and the recoveries percent were estimated from the respective regression equation.

#### Procedures for Human Plasma

One mL of CYZ was intravenously injected into three human volunteers in good health and Using a calibrated heparinized tube, a 5 mL sample of human blood was drawn from each volunteer after 0.5, 1, 2, 4, 8 hrs. All the collected samples were centrifuged for 20 min at 4000 rpm. 1 mL of each sample was quantitatively poured into a centrifuge tube, and 1 mL acetonitrile was applied to precipitate plasma proteins, until centrifugation at 4000 rpm for 20 minutes. The clear plasma supernatant was transferred to Eppendorf tubes and held at 20 °C until the rest of the procedure was completed.

#### Calibration Standards and Quality Control Samples in Pure Form

A series of 10 mL volumetric flasks were filled with accurately calibrated aliquots of CYZ working standard solution involving the range of experimental concentrations were poured into a series of 10 mL volumetric flask, with final concentrations varying from 10.0-1000.0 ng/mL using acetonitrile as diluting solvent. Concentrations of CYZ calibration standard were: 10, 20, 30, 50, 100, 200, 300, 400, 500, 800, and 1000 ng/mL. While, the concentrations of quality control sample were: 50, 400, and 800 ng/mL.

#### Calibration Standard and Quality Control Samples in Human Plasma Samples

Five mL of drug-free human blood was drawn from healthy volunteers and centrifuged at 4000 rpm for 20 min into a heparinized tube. Then, into 10-mL stoppered calibrated tube, in a 10-mL centrifuge tube, 1.0 mL of drug-free plasma sample was spiked with 0.1 mL CYZ stock solution (1.0 mg/mL). The fillings were then vortexed for 2 minutes before being finished with acetonitrile to precipitate the protein content and centrifuged at 4000 rpm for 20 minutes. The clear supernatant was drawn (working spiked plasma solution, 10.0 μg/mL), and then the detailed steps as below general analytical method were tracked. The same measures were followed for a blank, but without spiking 0.1 mL CYZ. Different CYZ concentrations in the range of 10.0-1000.0 ng/mL were made from its working spiked plasma solution (10.0 μg/mL) in a set of 10-mL volumetric flasks. Each flask received 1.0 mL of blank plasma, which was then filled with acetonitrile to make up the volume. The solutions were vortexed for one minute and centrifuged at 4000 rpm for 20 minutes. The supernatant was moved to a new series of 10-mL volumetric flasks, dried, and then reconstituted in 2 mL acetonitrile before proceeding with the general analytical method.

#### Analysis of Laboratory-prepared Mixtures

Different mixtures containing various ratios of CYZ and its official impurities were correctly moved from their respective working standard solutions into a set of 10-mL volumetric flasks and volumes were finished till the mark with acetonitrile. To investigate the stability of CYZ in the presence of its pharmacopeial impurities MPZ and DPM, the fluorescence intensity was measured at 350 nm after excitation at 244 nm. A blank was conducted at the same time.

## Results and Discussion

**Fig. 2:**
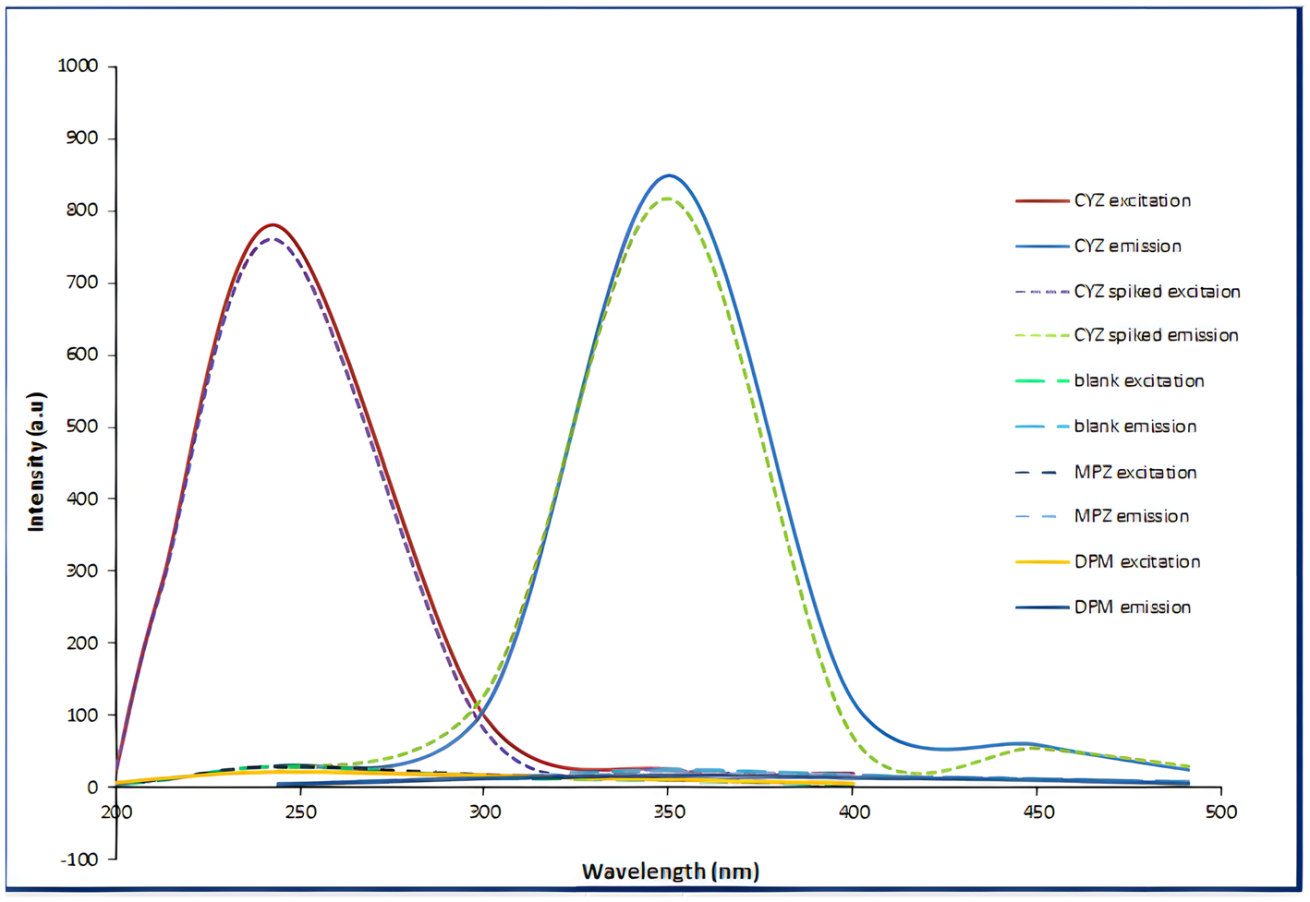
Fluorescence spectra of 1000 ng/mL of CYZ in pure form and in spiked plasma after using blank

Spectrofluorimetric method has many advantages like great sensitivity, selectivity, reduced light-scattering interference, rapidity, easiness of spectral complexity, and quite low prices [26]. Additionally, it has a linear response over a broad concentration range and high flexibility, owing to the ability to choose both the emission and excitation wavelengths, and to analyze samples in both solid and liquid states [30]. Therefore, the aim of this research was to develop a simple, low-cost, and extremely sensitive spectrofluorimetric procedure to determine CYZ in its purified form, pharmaceutical dosage form, and human plasma. CYZ had a native fluorescence in acetonitrile at 350 nm after excitation at 244 nm, as shown in Fig. 2. The proposed method was the first developed spectrofluorimetric quantification of CYZ with good selectivity and sensitivity based on the measurement of CYZ's native fluorescence after excitation with a wavelength that produces the extreme excitation intensity.

### Optimization of the Experimental Parameters

All variables that affect the spectrofluorimetric method were examined and optimized to enhance the fluorescence intensity which includes: diluting solvent, surfactant, plasma protein precipitating solvent and buffer pH.

#### Effect of Diluting Solvents

**Fig. 3:**
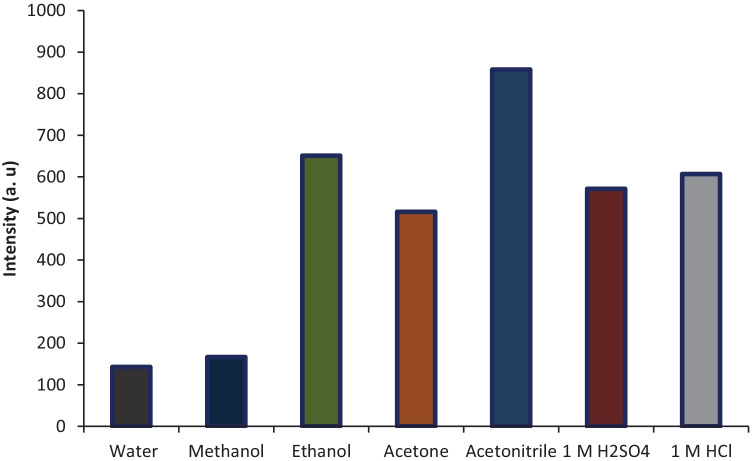
Effect of different diluting solvents on intensity of 1000 ng/mL of CYZ

The influence of various solvents on the fluorescence intensity was examined. Dilution with different solvents like distilled water, methanol, ethanol, acetone, acetonitrile, 1 M H_2_SO_4_ and 1 M HCl shown that acetonitrile was the finest solvent to use, as it produced the greatest relative fluorescence intensities and the smallest blank readings with consistent results Fig. 3. Consequently, acetonitrile was used throughout this analysis with extreme excitation and emission intensities at wavelengths 244 and 350 nm, respectively.

#### Effect of Surfactant

**Fig. 4:**
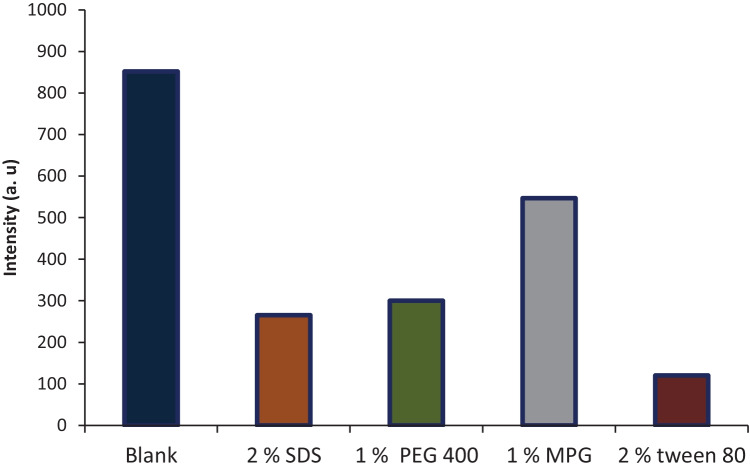
Effect of type of surfactant including sodium dodecyl sulfate (SDS), poly ethylene glycol (PEG), monopropylene glycol, and tween 80 on RFI of 1000 ng/mL of CYZ

Various surfactants were used comprising: 1% w/v MPG 400, 1% w/v PEG 400, 0.02 M SDS, and 2% v/v tween 80 in an attempt to enhance the relative intensity of CYZ in acetonitrile. It was obvious from the results shown in Fig. 4 that SDS, PEG 400, and MPG 400 decreased fluorescence intensity compared to the blank, which was acetonitrile without any surfactant, but with tween 80 this quenching was dramatic. Thus, no surfactant was used in this study as it had a negative effect on CYZ fluorescence intensity.

#### Effect of Plasma Protein Solvent

Plasma protein precipitation is a critical step to separate the protein from the solution to eliminate endogenous interferences in plasma samples to determine CYZ in human plasma. Hence, many precipitating solvents including methanol alone, acetonitrile alone, methanol and acetonitrile mixture (1:1 v/v ratio) were examined to attain full precipitation of plasma proteins in human plasma samples. Only acetonitrile was found to provide full precipitation of plasma proteins with a clear layer.

#### Effect of pH

**Fig. 5:**
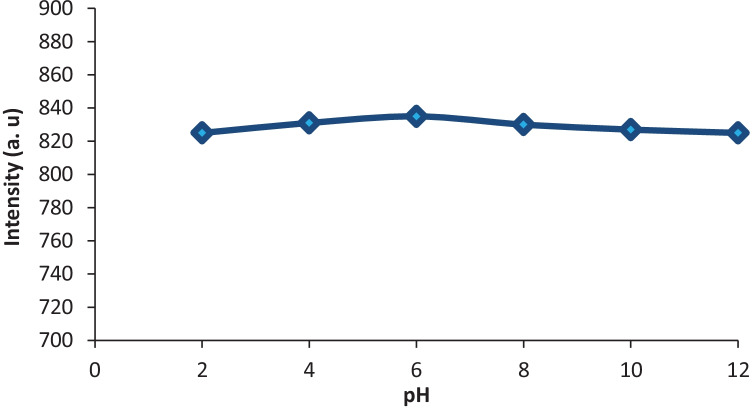
Effect of pH on RFI of 1000 ng/mL of CYZ

The pH effect on CYZ fluorescence intensity was tested in wide pH range of 2–12 by means of disodium hydrogen phosphate solutions which adjusted their pH values using phosphoric acid and sodium hydroxide. It was observed that, the intensity of CYZ is not significantly influenced by pH alteration in the considered range, Fig. 5. Consequently, the suggested procedure did not include the use of a buffer solution.

### Method Validation

The established procedure was well validated in accordance with the guidelines of ICH [31] and FDA bioanalytical method validation [32].

#### Linearity and Range

A linear relationship was found between emission intensities (FI values) and corresponding CYZ concentrations in the range of 10.0–1000.0 ng/mL under the defined experimental conditions for a calibration graph constructed either in pure or in spiked human plasma preparations. Linearity of CYZ relating the established method was reached to 0.9999 as a brilliant correlation coefficient. The found parameters of regression equation clarified in Table 1. The lowermost concentration on the calibration curve created in human plasma was established as the LLOQ. The back-calculated concentration had to have precision within 20% of the coefficient of variation (CV) and accuracy within 20% of the nominal concentration to pass the acceptance criterion. The LLOQ was determined using six samples and was 10 ng/mL with a CV % 1.89.

**Table 1:** Analytical parameters for analysis of CYZ by the proposed spectrofluorometric method

Parameter	Value
λex (nm)	244
λem (nm)	350
Linear range (ng/mL)	10–1000
Correlation coefficient (r)	0.9999
Intercept	37.935
Standard deviation of intercept	0.619
Slope	0.7974
Standard deviation of slope	0.010
Limit of detection (LOD, ng/mL)	3.10
Limit of quantitation (LOQ, ng/mL)	9.41

* LOD = 3.3 × SD/slope

** LOQ = 10 × SD/slope

#### Limit of Detection and Quantitation

The developed method had a great sensitivity and it can be used in detection CYZ in traces of biological fluids in accordance with ICH guidelines [31], LOQ and LOD values were calculated for CYZ to be 3.10 and 9.41 ng/mL, respectively. Whereas, according to FDA guidelines [32], LLOQ was evaluated to be 10 ng/mL.

#### Accuracy

Analyze six concentrations (20.0, 50.0, 100.0, 200.0, 400.0 and 800.0 ng/mL) of standard CYZ solution was performed to assess the accuracy by the proposed method. Using the regression equation, the concentrations were computed and the results were given in Table 2. Standard addition technique applied for further assessment of the accuracy of the developed method as illustrated in Table 3.

**Table 2:** Accuracy of the proposed method at six concentration levels within the specified range

Taken (ng/mL)	Found ± SD (ng/mL)	% Recovery ± SD
20	20.15 ± 0.12	100.73 ± 0.25
50	50.87 ± 0.98	101.74 ± 0.97
100	101.66 ± 1.10	101.66 ± 0.89
200	203.24 ± 1.54	101.62 ± 1.09
400	395.12 ± 3.54	98.78 ± 1.12
800	791.40 ± 6.43	98.93 ± 1.32

**Table 3:** Results of determination of CYZ in pharmaceutical formulation by the proposed method

**Emetrex**^**®**^** ampules** **Each ampules claimed to contain** **50 mg CYZ (Batch NO. 182541 A)**	**CYZ**	**Taken** **(ng/mL)** 200	% Found^*^ **± SD** 96.71± 1.45	**Standard addition technique**		% Recovery^**^
				**Pure added** **(ng/mL)**	**Pure found** **(ng/mL)**	
				100	100.33	100.33
				200	203.16	101.58
				400	407.58	101.90
	**Mean ± SD**			101.27 ± 0.83		

^*^: Average of six determinations

^**^: Average of three determinations

#### Precision

Intra-day precision of the proposed procedure was assessed by examining three different concentration levels of the standard CYZ solution (50.0, 400.0 and 800.0 ng/mL) within the same day. While the inter-day precision was evaluated by analyzing these concentrations for three consecutive days by applying the general analytical procedure. Following that, concentrations were determined using the earlier computed regression equation, and good % RSD values were obtained and given in Table 4.

**Table 4:** The intra- and inter-day precision for determination of CYZ by the proposed spectrofluorometric method

Concentration (ng/mL)	%Recovery* ± RSD	
	**Intra-day precision**	**Inter-day precision**
**Standard drug solutions**		
50	99.78 ± 1.88	98.53 ± 1.88
400	98.00 ± 0.78	97.45 ± 0.24
800	98.22 ± 0.71	97.83 ± 1.10
**Spiked rat plasma**		
30	97.90 ± 1.25	97.90 ± 2.51
400	97.37 ± 1.41	96.58 ± 1.25
800	97.28 ± 0.55	96.89 ± 0.63

* the values are the average of three determinations

#### Selectivity

Selectivity of the established method was considered through detection of CYZ in its parenteral formulations (Emetrex^®^ ampules). The percent recovery was assessed and good results were achieved 96.71± 1.45 as in Table 3. Moreover, Selectivity of the process was inspected by its capability of measuring CYZ in presence of its pharmacopeial impurities and no interference was attained by quantifying the fluorescence intensity at 350 nm after excitation at 244 nm for different laboratory mixtures with different ratios.

#### Stability of CYZ

To cover the anticipated sample treatment and storage environments during the processing of samples, the stability of CYZ in human plasma was investigated as short‐term stability at room temperature and freeze–thaw stability. In all stability tests, three replicates of three concentrations at low, medium, and high levels were treated and compared to recently prepared QC samples. All of the stability sample findings met the stability study's acceptance requirements according to FDA guidelines [32], Table 5.

**Table 5:** Stability of CYZ in rat plasma at different conditions by the proposed spectrofluorimetric method

Concentration (ng/mL)	% Remaining*	
	**Room temperature for 6 h**	**Three freeze thaw cycle**
30	96.98 ± 1.13	97.14 ± 1.32
400	98.03 ± 0.98	96.50 ± 0.97
800	96.13 ± 1.88	97.25 ± 1.76

* The values are the mean of three determinations

### Analysis of pharmaceutical dosage form

Under the previously described general analytical method, Emetrex^®^ ampules were examined. Appropriate percentage recoveries and standard deviations of 96.71± 1.45 were obtained, which judged statistically with the findings achieved by employing the reported HPLC method [20] for student t- and F- tests. As in Table 6, the results were found to be lower than those of the reported method, which indicated that there was no important alteration in precision and accuracy between the developed method and the reported one.

**Table 6:** Statistical comparison of the results obtained by applying the proposed method and the reported HPLC method for determination of CYZ in its pharmaceutical injection

	**Method**	**Spectrofluorimetric method**	**Reported** **Method** [20]^**^
	**Parameters**		
**Emetrex**^**®**^** ampules**	Mean* (%)	96.71	96.79
	**S.D**	1.45	1.24
	**N**	6	6
	**Student’s** ***t*-test** **(2.228)**^**a**^	0.11	-----------
	***F*-test** **(5.505)**^**a**^	1.41	-----------

* The values are the mean of six determinations

** Reported method is HPLC for determination of CYZ using acetonitrile- 0.05 M (pH = 4) phosphate buffer (50: 50, v/v/), as the mobile phase using Shim-pack RP C18 column (250×4.6 mm) with UV detection at 239 nm

### Analysis of biological samples and PK calculations

**Fig. 6:**
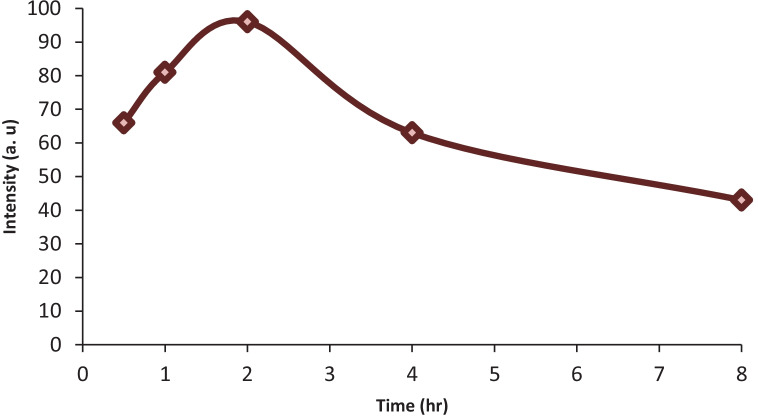
Mean plasma concentration-time plot of CYZ after intravenous administration (Emetrex^®^ 50 mg/mL ampules) to human volunteers by the proposed spectrofluorimetric method

Non-compartmental analysis (NCA) was used to measure pharmacokinetic parameters such as half-life, C max, T max, elimination rate constant, clearance, apparent volume of distribution, and area under the curve using Thermo Kinetica software (version 5.0) and PK solver software after taking human plasma samples from previously injected volunteers. In deciding the proper usage of the drug and in forecasting the impact of pharmacokinetic drug interactions, the results of pharmacokinetic research have been helpful. The C max of CYZ was measured and found to be 72.82 ± 1.56 ng/mL, while the T max was also assessed and found to be 2 hr. Moreover, other pharmacokinetic parameters like clearance, area under the curve, elimination rate constant, and apparent volume of distribution were efficiently computed as presented in Table 7, and Fig. 6 displayed the average plasma concentration-time profiles (n=7) of CYZ. The developed method showed a good similarity in the values of the calculated pharmacokinetic parameters with the reported values [15], consequently, such approach verified to be equivalent concerning precision and accuracy.

**Table 7:** Pharmacokinetic parameters of CYZ in human plasma after I.V administration of Emetrex® 50 mg

Parameter	Value
t_1/2_(h)	2.53 ± 0.43
C max (ng/mL)	72.82 ± 1.56
T max (h)	2.00 ± 0.21
Elimination rate constant (K)	0.274 ± 0.10
Volume of distribution (V_d_) (L)	0.695 ± 0.23
Clearance (cl) (L/h)	0.0002 ± 0.001
AUC _0→t_ (µg/mL.h)	22.30 ± 1.22
AUC _0→∞_ (µg/mL.h)	213.14 ± 7.21

### Prediction of the Pharmacokinetic Properties and Toxicological Properties Using ADMET

In this work, pkCSM software was used to forecast the toxicological characters of CYZ impurities MPZ and DPM under study which did not have native fluorescence. Furthermore, this software has a framework that conducts expectations of the kind of toxicity that a compound exhibits, such as hepatotoxicity, mutagenicity, and cardiotoxicity. Therefore, the pharmacokinetic predictions of the CYZ pharmacopeial impurities were anticipated. It is necessary to obtain human pharmacokinetic information for CYZ pharmacopeial impurities for the first time to appraisal the absorption, distribution, metabolism, and excretion of investigational impurities in healthy volunteers. Additionally, prediction the toxicological profiles for MPZ and DPM can assist in estimating the significance of analysis of the proposed drug in presence of its impurities and detect the critical limits for levels of impurities in the studied drug.

The findings showed that all the studied impurities showed significant values for oral absorption. The two impurities are soluble in water, but DPM is more water soluble than MPZ. High cellular permeability, especially for intestinal cells (96.311 and 93.762 %), is expected for both impurities.

The Caco-2 cell line is comprised of human epithelial colorectal adenocarcinoma cells. As an in vitro model of the human intestinal mucosa, the Caco-2 monolayer of cells is widely used to expect the absorption of orally administered drugs. Therefore, the studied impurities showed high measured permeability using the human colon adenocarcinoma (Caco-2) cell line assay [33]. MPZ anticipated to be substrates of P-glycoprotein, which is a member of the ATP-binding cassette transporter located mainly in epithelial cells.

A significant parameter to remember is the capacity of the compound to cross into the brain to help minimize side effects and toxicity. Expectation of the distribution possessions indicated that DPM has the uppermost blood–brain barrier (BBB) and CNS permeability other than MPZ. While, MPZ has higher unbound fraction and distribution volume than DPM.

DPM presented comparatively high action as they hinder CYP1A2, CYP2C19, and CYP2C9. This can be optimistically interrelated to the lipophilicity of the compound to metabolism related toxicity. These findings indicate that DPM can play a role in drug–drug interactions as well as oxidative stress. According to excretion, MPZ exhibited a higher total clearance comparative to DPM.

Concisely, DMP has elevated toxicity in *T. pyriformis* which is a protozoal bacteria and its toxicity often used as a toxic endpoint. The investigated impurities showed high maximum tolerated doses. Additionally, oral rat acute toxicity (LD50) was examined to assess the relative toxicities of the studied impurities as the lethal dosage values (LD50) are a typical measurement of acute toxicity, the obtained results shown in Table 8.

**Table 8:** ADMET properties of the CYZ two impurities

**Property**	**Items**	**MPZ**	**DPM**	Reference^*^[33]
**Absorption**	Water solubility (log mol/L)	0.51	-3.142	Solubility increased by decreasing log S
	CaCO-2 permeability (log Papp in 10^-6^ cm/ S)	1.334	1.728	High permeability > 0.90
	Intestinal absorption (%)	96.311	93.762	High absorbed > 30%
	P-Glycoprotein substrate	Yes	No	
**Distribution**	VDss (log L/Kg)	0.756	0.364	Low ˂ -0.15 High > 0.45
	Fraction unbound (Fu)	0.9	0.095	
	BBB permeability (log BB)	0.01	0.346	Log BB ˂ -1 poorly distributed to the brain Log BB > 0.3 cross the BBB
	CNS permeability (log PS)	-2.299	-1.618	Log PS ˂ -3 unable to penetrate CNS Log PS > -2 penetrate CNS
**Metabolism**	CYP1A2 inhibitor	No	Yes	This can be positively correlated to the lipophilicity of the compound to metabolism related toxicity
	CYP2C19 inhibitor	No	Yes	
	CYP2C9 inhibitor	No	Yes	
**Excretion**	Total clearance (log mL/min/Kg)	0.934	0.157	
	Renal OCT2 substrate	No	No	
**Toxicity**	Max. tolerated dose (log mg/kg/ day)	1.071	0.664	Low ≤ 0.477 High > 0.477
	Oral rat acute toxicity (LD50) (mol/kg)	1.947	1.891	
	Hepatotoxicity	No	No	
	T. pyriformis toxicity (log µg/L)	-0.687	1.322	Not toxic ˂—0.5 Toxic >—0.5
	Minnow toxicity (log mM)	3.232	1.06	Highly acute toxic ˂—0.3 Not highly acute toxic >—0.3

^*^Reference values of the pKCSM pharmacokinetics predictions properities

## Conclusion

The present project introduces for the first time spectrofluorimetric approach for detection of CYZ in its commercial parenteral dosage forms and human plasma based on its intrinsic fluorescence. It is extensively sensitive than most of other published methods. Additionally, it poses rapid response, simplicity, and low charge. Besides, the developed method has been applied successfully for prediction the pharmacokinetic behavior of CYZ in human plasma and its impurities by using either non-compartmental analysis (NCA) or computer aided software for in silico study and ADMET predictions, respectively.

## Data Availability

The data are available from the corresponding author upon sensible request.

## Declarations

### Source of Biological Material

All the used biological samples were taken from human volunteers after taken their verbal approval and according to Ethical approval guidelines.

## References

[1] British pharmacopoeia (2018) Vol. 1. London: Medicines and Healthcare products Regulatory Agency 633–634

[2] Arnestad M, Beate K, Eldor B, Stray-pedersen A, Bachs L, Karinen R (2014). Suicide Due to Cyclizine Overdose. J Anal Toxicol.

[3] Woodfield J, George EJS (2013). Post-operative cyclizine misuse. Scottch. Med J.

[4] Bailey F, Davies A (2008). The misuse/abuse of antihistamine antiemetic medication (cyclizine) by cancer patients. Palliat Me.

[5] De Silva AN, Stroud MA, Fine DR (2008). Cyclizine dependence in patients with complex nutritional requirements. Proc Nutr Soc.

[6] Bassett KE, Schunk JE, Crouch BI (1996). Cyclizine abuse by teenagers in Utah. Am J Emerg Med.

[7] Scientific T (2019) Material Safety Data Sheet of N-Methylpiperazine

[8] Scientific T (2019) Material Safety Data Sheet of Benzhydrol

[9] Saad AS, Naguib IA, Draz ME, Zaazaa HE, Lashien AS (2018). Validated analytical methods for the determination of drugs used in the treatment of hyperemesis gravidarum in multiple formulations. J AOAC Int.

[10] Habib NM, Abdelwhab NS, Abdelrahman MM, Ali NW (2016). Spectrophotometric methods for analysis of different dosage forms containing pyridoxine hydrochloride. Eur J Chem.

[11] Al-shaalan NH (2012). Extractive spectrophotometric assay of cyclizine in a pharmaceutical formulation and biological fluids. Saudi Pharm J.

[12] Abdelrahman MM, Fares MY, Abdelwahab NS, Hegazy MA, El-Sayed GM (2020). Ecofriendly validated chromatographic methods for quantitation of cyclizine and its toxic impurities in its parenteral formulation. Chromatographia.

[13] Habib NM, Abdelrahman MM, Abdelwhab NS, Ali NW (2017). Validated chromatographic methods for the analysis of two binary mixtures containing pyridoxine hydrochloride. J AOAC Int.

[14] Wolff K, Sanderson MJ, Hay AWM (1990). A rapid horizontal TLC method for detecting drugs of abuse. Ann Clin Biochem.

[15] Walker RB (1995) HPLC Analysis and Pharmacokinetics: no. 1

[16] Packert B, Winifred J, Vella-brincat A, James E (2011). Quantification of cyclizine and norcyclizine in human plasma by liquid chromatography – tandem mass spectrometry ( LC – MS / MS ). J Chromatogr B.

[17] Mohammadi A, Kanfer I, Sewram V, Walker RB (2005). An LC – MS – MS method for the determination of cyclizine in human serum. J Chromatogr B.

[18] Mao Y, Carr PW (2001). Separation of selected basic pharmaceuticals by chromatography using thermally tuned tandem columns. Anal Chem.

[19] Jonczyk A (1999). Determination of cyclizine hydrochloride, caffeine and ergotamine tartarate mixtures by high performance liquid chromatography (HPLC). Acta Pol Pharm Drug Res.

[20] El-gindy A, Emara S, Mostafa A (2004). Spectrophotometric and LC determination of two binary mixtures containing antihistamins. Farm.

[21] Mohammadi A, Kanfer I, Walker RB (2004). A capillary zone electrophoresis (CZE) method for the determination of cyclizine hydrochloride in tablets and suppositories. J Pharm Biomed Anal.

[22] Sarma BK, Rani S (2017). Analytical determination of cyclizine by using ion selective electrodes. Asian J Pharm Pharmacol.

[23] Rani S, Singh G (2016). Ion selective electrodes for potentiometric determination of cyclizine in its pharmaceutical dosage form. Asian J Pharm Sci Technol.

[24] Raghu PS, Sarma BK, Rani S, Javvaji MK (2018). Ion selective electrodes for potentiometric determination of cyclizine in its pharmaceutical dosage form. J Glob Trends Pharm Sci.

[25] Ganjali MR, Larijani B, Faridbod F, Norouzi P (2013). Potentiometric determination of cyclizine by a PVC membrane sensor. Int J Electrochem Sci.

[26] Andrade-Eiroa A´, Estela M, De-Armas G, Cerda `V (2010). Critical approach to synchronous spectrofluorimetry. Trends Anal Chem.

[27] Elzanfaly ES, Amer EA, Galal SAB, Zaazaa HE (2019). Spectrofluorimetric determination of eptifibatide in human plasma and dosage form. Luminescence.

[28] Arnold K, Bordoli L, Schwede T (2006). The Swiss-Model Workspace : A web-based environment for protein structure homology modelling Structural bioinformatics. Bioinformatics.

[29] Cheng F, Liu G, Tang Y (2020). In silico ADMET prediction : Recent advances, current challenges and future. Curr Top Med Chem.

[30] Prodi L, Credi A (2012). Spectrofluorimetry. chapter.

[31] ICH, Harmonised Tripartite Guideline (2005) Validation of Analytical Procedures: Text and Methodology Q2 (R1)

[32] FDA (2013) Guidance for Industry Bioanalytical Method Validation Guidance for Industry Bioanalytical Method Validation. https://www.fda.gov/downloads/drugs/guidances/ucm386366.pdf

[33] Pires DEV, Blundell TL, Ascher DB (2015). pkCSM : predicting small-molecule pharmacokinetic properties using graph-based signatures. J Med Chem.

